# Systematic identification of regulatory variants associated with cancer risk

**DOI:** 10.1186/s13059-017-1322-z

**Published:** 2017-10-23

**Authors:** Song Liu, Yuwen Liu, Qin Zhang, Jiayu Wu, Junbo Liang, Shan Yu, Gong-Hong Wei, Kevin P. White, Xiaoyue Wang

**Affiliations:** 1 State Key Laboratory of Medical Molecular Biology, Department of Biochemistry and Center for Bioinformatics, Institute of Basic Medical Sciences, Chinese Academy of Medical Sciences, School of Basic Medicine, Peking Union Medical College, Beijing, 100005 China; 2 Institute for Genomics and Systems Biology, and Department of Human Genetics, University of Chicago, Chicago, Illinois 60637 USA; 3 Biocenter Oulu, Faculty of Biochemistry and Molecular Medicine, University of Oulu, FIN-90014 Oulu, Finland; 4 Tempus Labs, Inc., Chicago, Illinois 60654 Finland

**Keywords:** GWAS, STARR-seq, Regulatory variants, Cancer susceptibility, CRISPR interference

## Abstract

**Background:**

Most cancer risk-associated single nucleotide polymorphisms (SNPs) identified by genome-wide association studies (GWAS) are noncoding and it is challenging to assess their functional impacts. To systematically identify the SNPs that affect gene expression by modulating activities of distal regulatory elements, we adapt the self-transcribing active regulatory region sequencing (STARR-seq) strategy, a high-throughput technique to functionally quantify enhancer activities.

**Results:**

From 10,673 SNPs linked with 996 cancer risk-associated SNPs identified in previous GWAS studies, we identify 575 SNPs in the fragments that positively regulate gene expression, and 758 SNPs in the fragments with negative regulatory activities. Among them, 70 variants are regulatory variants for which the two alleles confer different regulatory activities. We analyze in depth two regulatory variants—breast cancer risk SNP rs11055880 and leukemia risk-associated SNP rs12142375—and demonstrate their endogenous regulatory activities on expression of *ATF7IP* and *PDE4B* genes, respectively, using a CRISPR-Cas9 approach.

**Conclusions:**

By identifying regulatory variants associated with cancer susceptibility and studying their molecular functions, we hope to help the interpretation of GWAS results and provide improved information for cancer risk assessment.

**Electronic supplementary material:**

The online version of this article (doi:10.1186/s13059-017-1322-z) contains supplementary material, which is available to authorized users.

## Background

Genome-wide association studies (GWAS) have been widely used to detect the single nucleotide polymorphisms (SNPs) associated with common traits and complex diseases [1]. Since 2005, more than 800 variants associated with risk of various types of cancer have been identified [1]. As with other complex diseases, more than 90% of the cancer susceptibility SNPs are not in protein-coding regions [1], making it difficult to decipher their functional impacts. Multiple mechanisms have been proposed for explaining how non-coding variants influence human disease, such as disrupting the splicing, translation, or stability of the protein-coding gene [2]. In addition to protein-coding genes, the risk-associated SNPs identified by GWAS also affect key noncoding genes for miRNAs and lncRNAs [3–5]. Recent studies have found that the GWAS SNPs reported to be associated with diverse phenotypes and diseases, and the SNPs in linkage disequilibrium (LD) with the reported ones, are enriched in open chromatin regions marked by DNase I hypersensitive sites (DHSs) and transcription factor (TF) binding sites [6–8]. Also, the GWAS SNPs are more likely to be in genomic loci associated with gene expression as identified by expression quantitative trait loci (eQTL) mapping [9, 10]. Therefore, it has been hypothesized that many GWAS variants exert their effects through modulating the transcriptional activities of genes controlled by the regulatory genomic elements in which they are located. Consistent with this hypothesis, several SNPs in enhancers have been identified to contribute to the risk of breast cancer, prostate cancer, or neuroblastoma by modulating the expression of critical cancer-associated genes [11–13].

Annotating cancer susceptibility SNPs using chromatin states, sequence motifs, and eQTL sites can help prioritize variants for further assessment on their functional consequences [14, 15]. To validate these predictions on a large scale, high-throughput experimental approaches to directly quantify their regulatory effects are urgently needed. Recent advances in synthetic biology and next-generation sequencing have enabled a dramatic increase in the throughput of the luciferase reporter assay, a well-established method for assessing transcriptional activities of genomic regulatory elements. By incorporating a unique DNA barcode for each testing sequence at the 3’ UTR of a reporter gene, the massively parallel reporter assay (MPRA) can simultaneously assess the transcriptional activities of several hundred thousand testing sequences based on the relative abundance of their corresponding barcodes in transcripts [16, 17]. At an even larger scale, the self-transcribing active regulatory region sequencing (STARR-seq) approach allows for directly measuring the activities of millions of enhancers by using testing sequences as their own reporters, taking advantage of the position-independent property of enhancers [18, 19]. These methods have the potential to be adopted for direct testing of regulatory SNPs. Recently, two groups have reported direct identification of expression-modulating variants associated with GWAS traits using modified MPRAs [20, 21]. They synthesized tens of thousands of DNA elements containing both alleles of each SNP to recapture the variants in a population to test by MPRA, with increased numbers of barcodes for each variant to improve the sensitivity and reproducibility [20, 21].

Here we report the use of a modified STARR-seq method to allow for large-scale, convenient, and direct testing of regulatory variants. We captured the naturally occurring population genetic heterogeneity in a STARR-seq screening library and transfected the library into HEK293T cells for regulatory activity measurement. We applied the method to analyze all the variants associated with cancer risk (10,673 SNPs linked with 996 cancer risk SNPs) and found 1333 SNPs in the genomic regions at 502 loci (50.4% of known cancer risk loci) with either positive or negative regulatory activities. Of these, 70 variants were observed to directly modulate transcriptional activities in an allele-specific manner for the elements where they are located. For two top-ranked regulatory variants, we also identified their target genes and validated their endogenous regulatory activities using targeted CRISPR interference (CRISPRi).

## Results

### A modified STARR-seq strategy to detect regulatory variants associated with cancer susceptibility

To detect regulatory variants associated with cancer risk, we focused on the 996 GWAS hits for cancer susceptibility and drug response catalogued in NHGRI up to 2013 [1]. As causal SNPs could be in LD with a SNP reported in the GWAS catalogue [7], we included 10,673 SNPs that were in high LD (r^2^ > 0.8) with the 996 reported SNPs (Additional file 1: Figure S1a). For each SNP, we designed capture probes targeting the 500-bp genomic region centered at the SNP. To maximize the representation of common SNPs, we captured genomic DNAs from ten individuals from a Chinese Han population. By simulation using Chinese Han population data in the 1000 Genomes Project, we found that over 96% of the common SNPs would be covered using DNA from ten individuals (Additional file 1: Figure S1b).

**Fig. 1:**
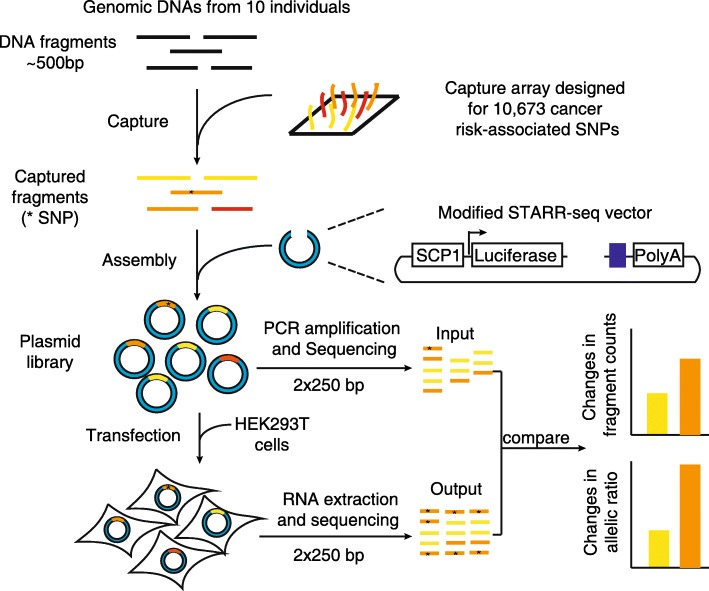
The workflow to screen for regulatory SNPs associated with cancer risk. The genomic DNA from ten individuals was pooled and sonicated into fragments of ~ 500 bp. Regions containing 10,673 SNPs in LD with 996 GWAS-identified cancer risk SNPs were captured using a custom designed array. The captured fragments were inserted into a modified STARR-seq vector using Gibson assembly to generate a plasmid library, which was sequenced as the input library and then transfected into HEK293T cells. The RNAs were extracted from cells and sequenced as the output library. The regulatory activities were calculated based on the ratio of normalized fragment counts in the output library against the input library. The regulatory SNPs were detected by the changes in allelic ratios in the output library compared to those in the input library

To directly detect the regulatory activity of the selected variants in a high-throughput way, we modified the STARR-seq strategy. We first rebuilt the pGL4.23 vector to have regulatory DNA fragments of interest inserted as self-transcribing elements in the 3’ UTR of the luciferase ORF (see “Methods”; Fig. 1). We replaced the pGL4.23 promoter with the SCP1 promoter and inserted a ccdB cassette to generate the vector pGL4.23-SCP1-ccdB [22–24]. The SCP1 promoter has been used in previous Starr-seq assays in mammalian cells and was validated to be able to accurately quantify enhancer activities [18, 19]. We also added an Illumina sequencing adapter to the vector right after an inserted element to simplify the construction of sequencing libraries.

The captured sequences were then amplified and inserted into our vector pGL4.23-SCP1-ccdB through Gibson assembly to generate the input plasmid library (Fig. 1). After transfection of the plasmid library, the mRNA was collected and the output library was prepared. We used 250-bp paired-end sequencing to ensure the detection of the variants at the fragment (Fig. 1). In this way, we may derive the allelic regulatory activities of a SNP by measuring the change of allelic ratios in the output library compared with those in the input library.

**Fig. 2:**
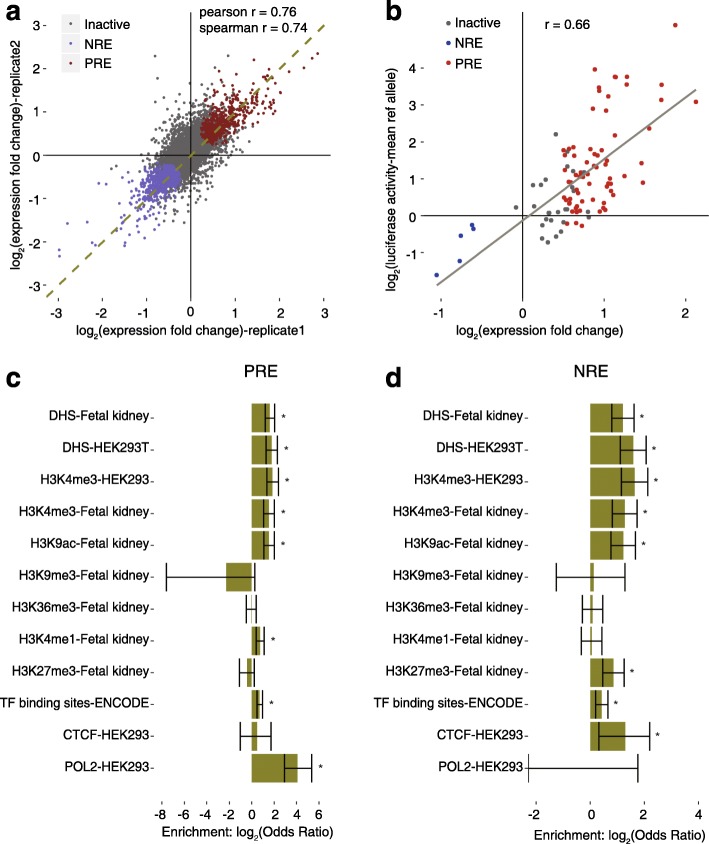
Regulatory regions identified in the screen and validation. **a** Correlation of the activities for the SNP-bound regions between two screens. The *p* value was calculated by Wald test, *p* value = 2.2 × 10^−16^. **b** Validation of identified enhancers using dual luciferase reporter assay; *r* represents Pearson’s correlation coefficient. The *p* value was calculated by Wald test, *p* value = 2.56 × 10^−14^. Identified positive regulatory regions (*PRE*) are in *red*, negative regulatory regions (*NRE*) are in *blue*, and inactive fragments are in *grey*. **c**, **d** Enrichments of epigenetic markers in the identified PREs and NREs, respectively. The *p* values were calculated by Fisher’s exact test; *p value < 0.05; *error bars* represent the confidence interval for the odds ratio

We observed high coverage of the designed SNP regions in the input library. From the raw reads of the two biological replicates, 97.3% of the designed SNP regions were recovered and 84% of them were sequenced at least ten times in both replicates, with a median depth of 204 and 175, respectively (Additional file 1: Figure S2a). In the output library, 99% of the fragments in the input library were recovered and 92.1% of the designed SNPs showed more than ten reads in both replicates (Additional file 1: Figure S2b). The normalized fragment counts in the input library were correlated with those in the output library for most SNPs. The outliers are likely to be the regulatory elements we are screening for (Additional file 1: Figure S2c). We also found that the screen is highly reproducible, as two transfection replicates performed in 293T cells were correlated with a Pearson coefficient of 0.99 (Additional file 1: Figure S2d). The calculated fold change for each fragment was also well correlated between two replicates (Fig. 2a).

### Regulatory activities for regions containing cancer risk GWAS SNPs

To determine the SNP-containing regions that have significant regulatory activities while accounting for the distribution of count data and sampling noise for fragments with low counts, we used DESeq2 [25] to calculate the fold change of normalized fragment counts from the output library over the input library from the data of two replicates (Fig. 2a; see “Methods” for details). According to DESeq2, 7725 SNP containing regions had sufficient counts for reliable testing for differences between the counts in the two libraries. Unlike previous MPRA studies in which a weak promoter was used, we found the distribution of expression fold change was not skewed toward the positive value (Additional file 1: Figure S2e), suggesting the potential to detect negative regulatory elements using a stronger promoter. With a false discovery rate (FDR) less than 0.01, we found 575 of the 7725 SNP-containing regions had a significantly increased count in the output library, while 758 of them had a significantly decreased count (Fig. 2a; Additional file 2: Dataset S1). We refer to these regions as positive regulatory elements (PREs) and negative regulatory elements (NREs), respectively. To validate the results of the screen, we tested the regulatory activities for 70 of the PREs, five of the NREs, and 27 inactive fragments using a classic luciferase reporter assay (Fig. 2b; Additional file 2: Dataset S2). The activities of these fragments in the luciferase assay were reasonably well correlated with the activities measured in our screen (Pearson correlation coefficient = 0.66), confirming the accuracy of the high-throughput assay in quantifying the regulatory activities.

Epigenetic marks, including DHSs, histone modifications, and transcription factor binding sites, are associated with genomic regulatory activity [8, 26]. To assess the endogenous chromatin features of the identified regulatory elements, we analyzed the available ENCODE data in HEK293 and HEK293T cells, as well as the Roadmap Epigenomics data in fetal kidney cells; 12.3% of the PREs and 9.23% of the NREs overlapped with DHSs in fetal kidney cells, while only 4.0% of the inactive fragments overlapped with DHSs (odds ratio [OR] = 3.08 for PREs and 2.31 for NREs, *p* value = 3.31 × 10^−13^ and 3.47 × 10^−8^, respectively, Fisher’s exact test; Additional file 3: Table S1). Similar enrichments were found for DHSs in 293T cells (OR = 3.46 for PREs and 3.01 for NREs, *p* value = 5.06 × 10^−11^ and 3.46 × 10^−10^, respectively, Fisher’s exact test; Additional file 3: Table S1). These results indicate that the regions of PREs and NREs are more likely to be within open chromatin and functional in endogenous contexts (Fig. 2c, d; Additional file 3: Table S1). The enrichment was also observed for marks associated with enhancers, such as H3K4me3 and H3K9ac (Fig. 2c, d). On the other hand, the epigenetic marks associated with heterochromatin (H3K9me3 [27]) and repressed transcription initiation (H3K36me3 [28]) were not enriched in either PREs or NREs (Fig. 2c, d). The differences in enrichments for specific histone marks between PREs and NREs may be explained by their opposite roles in regulating transcription. For example, the PREs are associated with H3K4me1, which marks cell type-specific “active” enhancers; while the NREs are associated with H3K27me3, the mark for Polycomb-mediated transcriptional silencing (Fig. 2c, d). Together these results suggest that the regulatory activities we observed, although identified using an ectopic assay, are mostly consistent with their transcriptional regulatory functions in the native genomic context.

As expected, both PREs and NREs were enriched for TF binding sites in the ENCODE data for 91 cell lines (Fig. 2c, d). For specific TFs that have ChIP-seq data in HEK293 cells, the NREs were significantly overlapped with binding sites for CTCF, an architectural protein mediating interaction between transcription regulatory sequences [29]. These observations are consistent with the potential distal regulatory roles of the regulatory regions we have identified. Interestingly, the PREs were enriched in RNA polymerase II (POL2) binding sites while NREs were depleted of POL2 binding (Fig. 2c, d). Consistent with this, POL2 binding has been reported to be associated with active enhancers and responsible for transcribing enhancer RNAs [30], supporting the positive regulatory roles of PREs.

Each GWAS study could report multiple tag SNPs that are associated with cancer risk. To test whether the more confident SNP markers were more likely to be in the PREs and NREs than in the inactive regions, we included 28 GWAS studies reporting ten or more SNP markers each. In total, 443 tag SNPs reported in these studies were tested in our assay and 87 of them were found in PREs or NREs. We found an enrichment of the most significant SNP markers in functional regulatory elements, as 11 of the 28 tag SNPs with the lowest *p* value in each study were in PREs or NREs and the other 17 were in inactive regions (OR = 2.64, *p* value = 0.027, Fisher’s exact test).

We also identified that many SNPs in regions with regulatory activities were in LD with tag SNPs. Interestingly, 53.2% of the cases had more than one SNP linked with the same tag SNP in PREs or NREs (Additional file 1: Figure S3a). For 17.6% of them, both PREs and NREs are present in the same loci (Additional file 1: Figure S3a). The distances between PREs and NREs in the same loci have a median of 8741 bp (Additional file 1: Figure S3d), indicating that the PREs and NREs were unlikely to overlap in position. These results are consistent with the observations for GWAS loci in autoimmune disorders [31], in which multiple polymorphisms in LD could map to clusters of enhancer regions and might cooperatively impact gene expression.

### Identifying regulatory variants

We next focused on identifying the regulatory variants for which two alleles at the SNP site (reference and alternative alleles) showed different regulatory activities. With a fragment size of about 465 bp, we were able to robustly call the genotypes at each SNP position (Additional file 1: Figure S4a–c). By using genomic DNA from ten individuals from a Chinese Han population, we recovered 83.5% (8902 of 10,673) of the SNPs we attempted to capture in our experimental design, whereby both alleles were represented in our library. Applying the low coverage threshold in DESeq2 to eliminate SNPs with sparse data, we have included 7514 SNPs for further analysis. The allelic ratio for these SNPs in the input library was correlated with the allele frequency in the Eastern Asian population (Additional file 1: Figure S4d). We observed a strong correlation between the allelic ratios in the plasmid DNA library and the allelic ratios in the output library, indicating that most variants had only a small effect on regulatory activity (Additional file 1: Figure S4e).

**Fig. 3:**
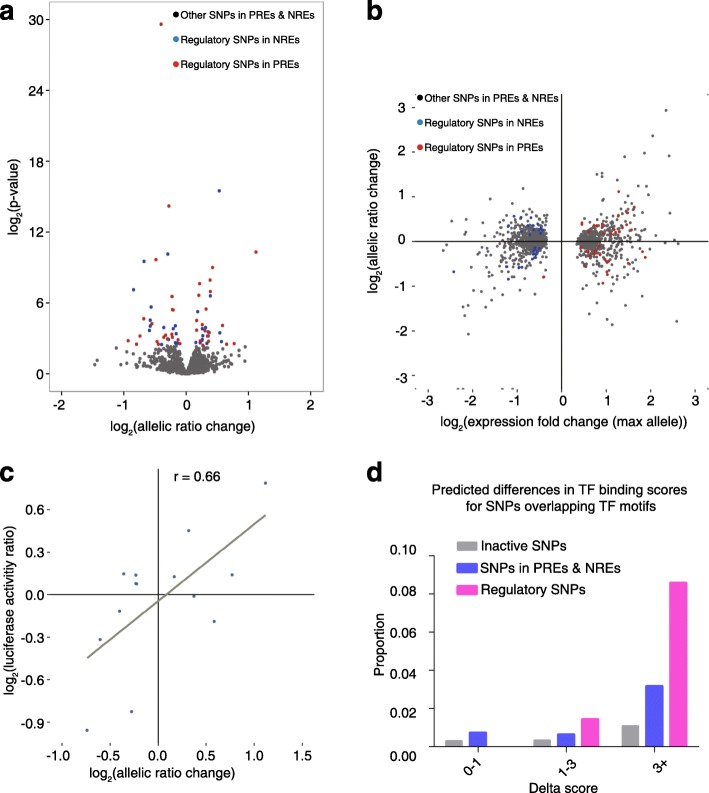
Identification and validation of regulatory SNPs. **a** Distribution of effect sizes and DESeq2 *p* values for all the SNPs that have two alleles covered. **b** Distribution of effect sizes of all the tested SNPs against the activities of the SNP-containing regions. The regulatory SNPs in PREs are shown in *red* and those in NREs in *blue*. **c** Luciferase reporter assay validation of the estimated effect sizes for 14 regulatory SNPs. *r* represents the Pearson correlation coefficient. **d** Differences in predicted TF binding scores between two alleles for different classes of SNPs

The imbalanced expression of two alleles in the output library compared to the input library was used to define regulatory variants and the statistical significance was evaluated by the two-sided Fisher’s exact test. The changes in allelic ratios were reproducible between two replicates (Additional file 1: Figure S4f). At a FDR < 0.1, we identified 70 SNPs with imbalanced expression of two alleles (Fig. 3a; Additional file 2: Dataset S1), 39 of which are in PREs and 31 in NREs. The change in allelic ratio was moderate for most sites and independent of the effect size of the fragment (Fig. 3b). We validated 14 of the 70 regulatory SNPs using a standard luciferase reporter assay and observed high correlation between the effect sizes of the two assays (Fig. 3c; Additional file 2: Dataset S2).

Similar to the overall set of PREs and NREs, these regulatory SNPs are enriched within transcription factor binding sites compared to inactive regions (OR = 2.08, *p* value = 7.5 × 10^−4^, Fisher’s exact test; Additional file 3: Table S2a). The regulatory SNPs that overlapped within a TF motif are also more likely to change the strength of TF binding than other SNPs. When we considered the number of variants that contributed a difference of at least 3 in log-likelihood binding score based on position-weight matrices, we observed 2.7-fold more variants in the regions showing allelic differences in expression compared to SNPs in regulatory sequences that did not show imbalanced allelic expression (OR = 2.7, *p* value = 0.0378, Fisher’s exact test); and we observed a 7.9-fold difference when compared to SNPs in inactive regions (OR = 7.9, *p* value = 2.2 × 10^−4^, Fisher’s exact test) (Fig. 3d; Additional file 3: Table S3).

eQTLs are often associated with *cis*-regulatory SNPs found in promoters and enhancers that contribute to differential gene expression. We found our regulatory variants were enriched in eQTL peaks identified from The Cancer Genome Atlas (TCGA) datasets of six cancer types (OR = 3.97, *p* value = 0.043, Fisher’s exact test; Additional file 4: Supplementary Text), suggesting they have endogenous expression modulating activities.

From the luciferase assay validation, we estimate our predictive positive value is about 57% (Fig. 3c). Based on the assumptions from previous MPRA studies, the sensitivity of our screen to identify a causal eQTL variant was between 10 and 12%, and the sensitivity of our screen to identify causal variants from GWAS hits was about 8.8% (Additional file 4: Supplementary Text).

### rs11055880 is a regulatory SNP in an intergenic enhancer for *ATP7IP* gene expression

**Fig. 4:**
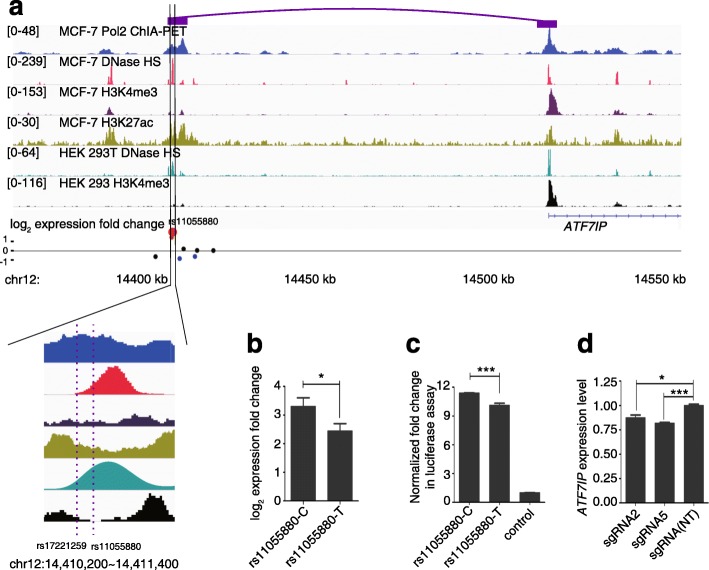
Regulatory SNP rs11055880 is in an intergenic enhancer regulating the expression of the *ATF7IP* gene. **a** Genomic context of rs11055880 shown in the integrative genome viewer. ChIA-PET signals in MCF7 cells (the interaction between rs11055880 and *ATF7IP* shown by the *purple boxes*), ENCODE annotations of DNase hypersensitive sites, H3K4me3, and H3K27ac in MCF7 cells, and DHSs and H3K4me3 marks in HEK293 cells are shown in tracks 1–6. The regulatory activities are shown in track 7. *Red dots* represent SNPs in PREs and the enlarged one is rs11055880. The *blue dots* represent SNPs in NREs and the *black dots* represent other tested SNPs in this region.  **b** Activities of two alleles of rs11055880 in our screen. Two-tailed paired *t*-test was used, *p value = 0.047. **c** Activities of two alleles of rs11055880 in the luciferase reporter assay. Two tailed t-test, ***p value = 2.0 × 10^−4^. **d** Expression levels of *ATF7IP* by qPCR in HEK293T cells expressing sgRNAs targeting the rs11055880 loci (rs11055880-sg2 and rs11055880-sg5) after KRAB-dCas9 activation. *P* values were calculated by *t*-test compared to a non-targeting (NT) group from three replicates; *p value = 0.016, ***p value = 4.0 × 10^−4^. For **b**–**d**, the error bars represent standard erorrs

After identifying 70 regulatory SNPs, we investigated several in greater detail. The first one we chose was rs11055880, which is located in one of the strongest PREs we have identified in the screen. It is in LD with rs17221259, a tag SNP reported to be associated with breast cancer in a GWAS of a Japanese population [32]. rs11055880 resides in DHSs in both MCF7 and HEK293T cells (Fig. 4a). It also overlaps with H3K4me3 peaks as well as H3K27ac marks, indicating endogenous enhancer activities for this region (Fig. 4a). In our assay, compared to the plasmid library, we found a 3.3-fold increase in expression for the fragment containing rs11055880-C and a 2.45-fold increase for rs11055880-T, which is a significant difference (Fig. 4b; n = 4, two tailed paired *t*-test, *p* value = 0.047). The difference in enhancer activity was validated using a luciferase reporter assay. After replacing the reference C allele with the alternative T allele, we observed the enhancer activity of the rs11055880 region reduced from 11.53-fold of the control to 10.32-fold (Fig. 4c; n = 6, two tailed *t*-test, *p* value = 2.0 × 10^−4^).

We next wanted to explore whether it is possible to identify potential targets of the rs11055880-containing region. The nearest gene is *ATF7IP*, 100 kb downstream of the SNP. In ChIA-PET data in MCF7 cells, we found an interaction of the SNP with the promoter of the *ATF7IP* gene (Fig. 4a). Consistent with this long-range interaction, in both GM12878 and IMR90 cell lines, rs11055880 and the *ATF7IP* gene were found together in one of the topologically associated domains (TADs) [33] (Additional file 1: Figure S5), the large local chromatin interaction domains defined by HiC data that are very stable across cell types [34, 35]. To validate that the rs11055880-containing region endogenously regulates *ATF7IP* expression, we used the CRISPR interference (CRISPRi) system to alter the chromatin state at the rs11055880 site through recruitment of a KRAB effector domain fused to catalytically dead Cas9 [36]. sgRNAs targeting the SNP region of rs11550880 resulted in a decrease of *ATF7IP* expression, consistent with our hypothesis (Fig. 4d). ATF7IP is a transcriptional cofactor that has been shown to be critical for heterochromatin formation by interacting with the histone methyltransferase SETDB1 [37], an oncogene product promoting tumorigenesis in melanoma, lung cancer, and liver cancer [38–40]. Therefore, the association of the rs11055880-containing locus with breast cancer susceptibility may be related to modulation of the expression levels of *ATF7IP*.

### The acute lymphoblastic leukemia risk-associated SNP rs12142375 modulates *PDE4B* gene expression

**Fig. 5:**
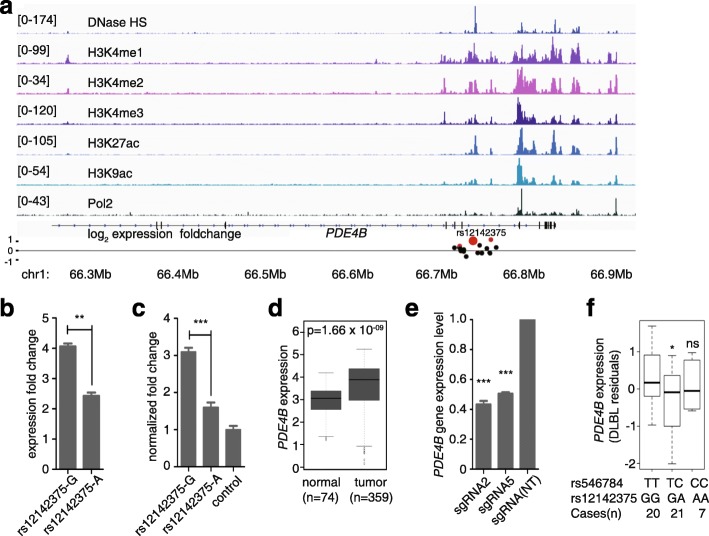
rs12142375 confers acute lymphoblastic leukemia risk mechanistically through modulating *PDE4B* gene expression. **a** Genomic map of the rs12142375 locus, with tracks of DNase I hypersensitive sites, H3K4me1, H3K4me2, H3K4me3, H3K27ac, H3K9ac marks, and Pol2 ChIP-seq signals in GM12878 cells. The *red dots* repesent the SNPs in PREs and the *black* *d* *ots* represent other tested SNPs in this region. rs12142375 is represented as the *big red dot*. **b** Two alleles of rs12142375 conferred different activities in our screen. Two-tailed *t*-test was used to calculate the *p* value, n = 4, **p value = 0.008. **c** Activities of two alleles of rs12142375 in the dual-luciferase reporter assay. The *p* value was calculated by two tailed *t*-test, n = 3, ***p value = 0.001. **d** *PDE4B* expression levels in peripheral blood mononuclear cells (normal, n = 74) and B cells of childhood acute lymphoblastic leukemia (tumor, n = 359) (data from the Haferlach Leukemia study). The *p* value was assessed by the Mann–Whitney U test. **e** Expression levels of *PDE4B* by qPCR in HEK293T cells expressing sgRNAs targeting the rs12142375 loci (rs12142375-sgRNA2, 24 bp upstream of the SNP, and rs12142375-sgRNA5, 11 bp downstream of the SNP) after KRAB-dCas9 activation. *P* values were calculated by Student’s *t*-test compared to the non-targeting (*NT*) group, n = 3, ***p value < 0.001. **f** eQTL results in TCGA diffuse large B-cell lymphoma dataset for the association of rs12141375 with *PDE4B* expression. The *p* value was calculated by one-tailed Student’s *t*-test, *p value = 0.023; *ns* not significant. For (**b**, **c**, **e**), the error bars represent standard errors

Among the regulatory SNPs that have the most distinct allele activities was rs12142375, which is in LD with a risk SNP identified in a GWAS of childhood acute lymphoblastic leukemia [41]. In lymphoblastoid cell line GM12878, rs12142375 was located within the DNase I hypersensitive site and a RNA polymerase II binding site. The rs12142375-containing region is also occupied by several histone marks of active enhancers such as H3K4me1, H3K4me2, H3K4me3, H3K27ac, and H3K9ac (Fig. 5a). Together, these ENCODE project data suggested an active enhancer role for the rs12142375-containing region in its native chromatin context. We then validated the enhancer activity of the region using a dual-luciferase reporter assay. Consistent with the result of the screen (Fig. 5b), the rs12142375-containing region with risk-associated allele G showed significantly higher enhancer activity than the region containing allele A (Fig. 5c). Next we aimed to explore the relationship between the regulatory SNP rs12142375 and the acute lymphoblastic predisposition. rs12142375 is located in the seventh intron of phosphodiesterase 4B (*PDE4B*), and about 15 kb far away from the nearest exon. *PDE4B* was reported to be highly expressed in CD4+ lymphoid cancer cells [42], with a role in promoting angiogenesis in B-cell lymphoma [43]. It also limits cAMP-associated PI3K/AKT-dependent apoptosis in diffuse large B-cell lymphoma [44]. By analyzing the *PDE4B* gene expression levels of cases with childhood acute lymphoblastic leukemia from microarray-based gene expression profiling [45], we also found that *PDE4B* was highly expressed in the cases (n = 359) compared to non-leukemia and healthy bone marrow (n = 74) (Fig. 5d; Mann–Whitney U test, *p* value = 1.66 × 10^−9^). To test whether the *PDE4B* expression was regulated by the rs12142375-containing enhancer, we used CRISPRi to inhibit the activity of the enhancer region. Indeed, the two sgRNAs targeting the rs12142375 region both significantly down-regulated *PDE4B* expression (Fig. 5e). To further test the allele-specific regulation of *PDE4B* expression by the SNP rs12142375 in B cells, we performed an eQTL analysis using the TCGA diffuse large B-cell lymphoma data. We inferred the genotypes of rs12142375 from the closely linked SNP rs546784 in the SNP array (r^2^ = 1). We observed that individuals with the GG genotype of rs12142375 have the highest expression of *PDE4B*, while individuals with heterozygosity genotype GA have significantly lower *PDE4B* expression levels (Fig. 5f; one-tailed Student’s *t*-test, *p* value = 0.026). We did not see a difference in *PDE4B* expression between the AA and GG genotype groups, probably due to a lack of statistical power with only seven individuals in the AA genotype group. Taken together, our results suggest that the association of rs12142375 with acute lymphoblastic leukemia risk might be due to a direct regulatory role of rs12142375 in *PDE4B* gene expression.

## Discussion

In this study, we have developed an approach to systematically screen for regulatory GWAS variants associated with cancer risk based on a modified STARR-seq method. Our assay robustly detected a range of expression changes between 0.5- and 16-fold, allowing the concurrent detection of elements with positive or negative effects on transcription. The 575 positive regulatory elements and 758 negative regulatory elements we identified are endogenously associated with regulatory markers such as TF binding, DHSs, specific histone modifications, and CTCF binding. Interestingly, a difference in endogenous POL2 binding was found between these two types of elements, indicating that POL2 binding may be used to differentiate active enhancers. Additionally, we found a group of GWAS variants that appear to modulate the transcription suppression role of certain DNA elements, which has not been reported before and warrants further investigation. Furthermore, widespread co-existence of positive and negative regulatory elements were observed in the same genomic loci in LD with individual GWAS tag SNPs, suggesting that gene expression associated with these regions may be regulated by multiple enhancers and silencers in a complicated way. Overall, our approach provided a convenient high-throughput method for directly testing the regulatory effects of GWAS variants, and serves as a complement for other MPRA approaches.

Based on the rate of detecting eQTLs, our assay has an estimated sensitivity between 10 and 12%, which is in line with the estimate that 23–64% of eQTLs act on promoters and enhancers [46]. Several factors could affect the sensitivity of our assay. First, some of the regulatory variants may be cell type-specific; the fact that we tested variants from GWAS studies of different cancer types, but only used one cell line, could limit the ability of detecting all variants. Second, for some of the SNPs showing allelic imbalance in expression, the numbers of reads covering the SNPs were lower than the threshold we set. Increasing the depth of sequencing, as well as the complexity of library, would increase the sensitivity in future studies.

Our assay also has a number of limitations. First, starting with DNA from ten different individuals, we recovered both alleles of the variants for only 84% of the designed SNPs. Although the distribution of the allele frequency in our library correlated well with that in the population, it is possible that we missed some rare SNPs that are more likely to exert larger functional impact. Future studies may overcome this latter limitation by including more samples at the start. In their study, Vockley et al. [47] used genomic DNA from a cohort of 95 people and had both alleles covered for 88% of the 104 candidate elements they selected, including rare variants with population frequencies less than 1%. Second, although we focused on identifying the transcription modulating regulatory elements, attaching the testing sequences after the luciferase inevitably results in discovery of other types of regulators, such as those affecting mRNA stability. Therefore, the screen could only be used to narrow down the candidates for transcription regulation but not fully demonstrate their modes of function. Nevertheless, when we validated the PREs and NREs we identified in the luciferase assay by inserting them before the start site of the reporter gene, we observed good correlation between the activities in the luciferase assay with those in the modified STARR-seq assay. These results suggest that changes in STARR-seq activities in our assay were likely primarily driven by the modulating effects on transcription rather than by any effects on post-transcriptional regulation affected by the expression of the regulatory element itself. Third, like other assays performed on cell lines, this study is limited in detecting cell type-specific regulatory elements. Here we chose HEK 293T cells as a proof of principle and the strategy could be easily adapted to different types of cell lines for studying tissue-specific enhancer variants.

In the future, it will be important to combine different methods, computational and experimental, to uncover the functional impacts of GWAS variants [48]. Our discovery of target genes for two of the regulatory variants demonstrated a first step in this direction. By combining the ENCODE ChIA-PET data and CRISPR-Cas9 technology, we were able to show that the strong intergenic enhancer covering the rs11055880 position endogenously regulates the expression of *ATF7IP*. We also validated the direct link between the risk-associated G allele at rs12142375 and increased *PDE4B* expression by eQTL analysis in clinical samples of B-cell lymphoblastic leukemia.

## Conclusions

We have developed a STARR-seq approach to systematically identify SNPs in both PREs and NREs of gene expression and, more importantly, to directly assess the impacts of the allelic change in SNPs on the regulatory activities of such elements. Applying the method to study the functional impacts of GWAS-identified cancer risk SNPs, we have uncovered 70 SNPs in regulatory elements with allele-specific activities on transcription. For two of them, we found their association with cancer risk may be explained by transcriptional regulation of cancer genes. Further studies on these regulatory variants will greatly improve our knowledge of cancer development and help develop better cancer risk assessment.

## Methods

### Design of the screen

#### Selection of cancer risk-associated SNPs

To select all the SNPs associated with cancer risk, we downloaded the publicly available GWAS catalogue data from the NHGRI website (http://www.genome.gov/gwastudies/, accession date 20150104). A total of 264 studies with the keywords matching at least one cancer type were included. All the tag SNPs with a significant association (*p* value < 10^−5^) were selected from these studies, resulting in 996 GWAS tag SNPs (Additional file 2: Dataset S1). SNPs in high linkage disequilibrium (LD) with the 996 SNPs were identified from the population matching the original GWAS using the HapMap project data (HapMap release #27). With the r^2^ set to 0.8, a total of 10,673 SNPs were defined as cancer risk-associated SNPs.

#### Construction of the new STARR-seq vector pGL4.23-SCP1-ccdB

To construct a modified STARR-seq vector for screen, the pGL4.23 (Promega, E8411) was first digested with HindIII and NcoI to remove the minimal promoter sequence. A synthesized Super core promoter 1 (SCP1) sequence (GTACTTATATAAGGGGGTGGGGGCGCGTTCGTCCTC AGTCGCGATCGAACACTCGAGCCGAGCAGACGTGCCTACGGACCG) was inserted into the digested pGL4.23 backbone using Gibson assembly. The CmR-ccdB suicide gene was PCR amplified from the STARR-seq vector (kindly provided by Dr. Alexander Stark) using primers containing the SphI-HF and the NdeI recognition site. It was then assembled with the linearized pGL4.23-SCP1 vector (digested by *FseI*) using Gibson assembly to generate the pGL4.23-SCP1-ccdB vector.

#### Genomic library preparation and capture

Human saliva was collected using a saliva DNA Sample Collection Kit (ZEESAN, 401002) and genomic DNA was isolated using a genomic DNA extraction kit (ZEESAN, 602001). Genomic DNA (1 μg) from each of the ten individuals of the Chinese Han population were pooled and sheared into ~ 500-bp fragments by sonication (Covaris S220). DNA fragments between 450 and 500 bp were size-selected on a 1.2% high-resolution agarose gel and recovered by TIANgel midi purification kit (TIANGEN, DP209). Recovered DNA fragments were analyzed by Bioanalyzer (Agilent) to validate the size distribution. End-repair and dA-tailing were performed with a NEBNext Ultra End Repair/dA-Tailing Module (NEB, E7442) with all recovered DNA fragments. Illumina multiplexing adapters were ligated to DNA fragments using a NEBNext Ultra Ligation Module for DNA (NEB, E7445) and purified with 1.2× Agencourt AMPure XP beads (Beckman, A63881). Adapter-ligated DNA fragments were amplified by PCR with amplification primers containing both illumina adapter sequences and homology arms with the vector (forward primer, GTAATAATTCTAGAGTCGGGGCGGGcatgAATGATACGGCGACCACCGAGATCTACACTCTTTCCCTACACGACGCTCTTCCGATCT; reverse primer, TATCATGTCTGCTCGAAGCGGCAtaGTGACTGGAGTTCAGACGTGTGCTCTTCCGATCT) using NEBNext® High-Fidelity 2× PCR Master Mix (NEB, M0541L) and purified with 1.2× Agencourt AMPure XP beads.

A custom Nimblegen capture system (Roche) was designed to capture the genomic regions from 250 bp upstream to 250 bp downstream of each of the 10,673 selected cancer risk-associated SNPs using the online NimbleDesign Software with the default settings (http://sequencing.roche.com/products/software/nimbledesign-software.html). The prostate cancer SNP rs339331 was included as a positive control. The capture was carried out according to the manufacturer’s instructions (SeqCap EZ Library SR User’s Guide, Nimblegen) starting with 1 μg DNA genomic library. We then amplified 50 μL of the captured DNA fragments in five independent 50-μL PCR reactions using NEBNext® High-Fidelity 2× PCR Master Mix (NEB, M0541L) with the amplification primers. The PCR products were pooled and purified with 1.2× AMPureXP DNA beads (Agencourt) for plasmid library cloning.

#### Cloning of plasmid library

The pGL4.23-SCP1-ccdB vector was linearized by double digestion with SphI-HF (NEB, R3182) and NdeI (NEB, R0111), and purified through electrophoresis and gel extraction. The captured DNA was cloned into the vector by mixing the DNA and linearized vector at a 5:1 ratio in 16 Gibson assembly reactions (NEB, E2611), each 20 μL. After purification, half of the assembled products were transformed into DH10B electrocompetent bacteria (Life Technologies, C6400-03) by electroporation using the default bacteria transformation setting of the electroporator (Biorad). After 1-h recovery at 37 °C in SOC, electroporated bacteria were split and plated to 80 LB plates supplemented with 100 μg/mL of ampicilin (Sigma-Aldrich, A9518) and grown overnight at 32 °C. Gradient dilute aliquots of the transformation were plated to estimate the size of the cloned library. The colonies were harvested by pipetting 10 mL of LB onto each plate and scraping the colonies off with a cell spreader. The plasmid library was then extracted using a Qiagen Plasmid Plus Mega Kit (Qiagen, 12981) and diluted to 1 μg/μL for all the following transfections.

To determine the sequences of the inserted DNA fragments, 1 ng plasmid library was amplified with PCR using primers AATGATACGGCGACCACCGAGATCTACACTCTTTCCCTACACGACGCTCTTCCGATCT (universal primer) and CAAGCAGAAGACGGCATACGAGATGATCTGGTGACTGGAGTTCAGACGTG (Illumina index 7 primer). The PCR products were purified using 0.8 × Agencourt AMPureXP DNA beads, quantified with an Agilent DNA1000 Chip (Agilent, 5067-1504), and then sequenced on a HiSeq 2500 (Illumina) with 250-bp paired-end sequencing.

#### Cell culture and plasmid library transfection

HEK293T cells were cultured in DMEM medium (Hyclone) supplemented with 10% heat-inactivated FBS (Gibco) at 37 °C. Library transfection was performed using the Neon Transfection System (Life Technologies). A total of 40 × 10^6^ cells were transfected. Each 5 × 10^6^ cells were suspended in 500 μL Buffer R (Life Technologies, MPK10096) with 30 μg library plasmids, then electroporated using conditions of 1260 V-20 ms-2pulses. Transfected cells were transferred to 10 mL pre-warmed growth medium and incubated for 24 h before RNA isolation.

#### RNA isolation and reverse transcription

Twenty-four hours post-electroporation cells were washed in 1 × PBS and harvested. Total RNA was extracted from all surviving cells using a Qiagen RNeasy maxi prep kit (QIAGEN, 75162), eluted with 1.5 mL nuclease-free water (Ambion, AM9938). The poly(A)-positive RNA was isolated using a Dynabeads mRNA Purification Kit (Life Technologies, 61006) following the manufacturer’s instructions. Then the mRNA was treated with TURBO DNase (Life Technologies, AM1907) for 30 minutes at 37 °C, followed by DNase inactivation and purification according to the kit protocol. Finally, the purified mRNA was quantified by NanoDrop 2000.

First strand cDNA synthesis was performed with SuperScript® III First-Strand Synthesis SuperMix (Life Technologies, 18080400) using a reporter RNA specific primer (5′ CAAACTCATCAATGTATCTTATCATG) and 450–500 ng mRNA per reaction for a total of 30 reactions. Five reactions were pooled (100 μL) and incubated at 37 °C for 1 h after adding 1 μL of 10 mg/mL RNaseA and 1 μL RNaseH (NEB, M0297).

#### cDNA amplification and sequencing

The cDNA was amplified in 120 PCR reactions (98 °C for 30 s, followed by 16 cycles of 98 °C for 10 s, 65 °C for 30 s, 72 °C for 30 s) using NEBNext® High-Fidelity 2X PCR Master Mix (NEB, M0541L), each started with 5 μL cDNA product with primers AATGATACGGCGACCACCGAGATCTACACTCTTTCCCTACACGACGCTCTTCCGATCT (universal primer) and CAAGCAGAAGACGGCATACGAGATTCAAGTGTGACTGGAGTTCAGACGTG (Illumina index 8 primer), or CAAGCAGAAGACGGCATACGAGATTACGTACGGTGACTGGAGTTCAGACGTG (Illumina index 22 primer). The PCR products were pooled and purified using 0.8× Agencourt AMPureXP DNA beads, eluted in 20 μL H_2_O, and quantified with an Agilent DNA1000 Chip (Agilent, 5067-1504). The output library was sequenced on an Illumina HiSeq 2500 using paired-end 250-bp reads.

### Data analysis

#### Simulation of SNP coverage with different numbers of individuals

Individual genotype data and sample information were downloaded from the 1000 Genomes Project (ftp://ftp.1000genomes.ebi.ac.uk/vol1/ftp/release/20130502/). The genotypes of the targeted 10,673 SNPs from the 98 Chinese Han individuals in Beijing (CHB cohort) and Southern Han Chinese (CHS cohort) were assembled as a pool. Different numbers (*i*) of individuals were randomly selected from the pool and the proportion of targeted SNP coverage at each sample size *i* was calculated as *P*_*i*_ = *N*_*i*_/10673, where *N*_*i*_ is the number of SNPs with both alleles covered. At each sample size *i*, the random sampling was repeated five times to calculate the standard deviation of *P*_*i*_.

#### Identification of positive and negative regulatory elements

The sequencing reads from two input libraries and two output libraries were mapped to the reference human genome (hg19) using BWA (version 0.7.12-r1039) [49]. Only fragments ranging from 400 to 600 bp and overlapping with at least one selected SNP were kept for further analysis. The fragment counts were normalized with a median-of-ratio method by DESeq2 [25]. For each SNP-containing fragment, the log_2_ fold change between the input library and the output library was calculated using DESeq2. Wald’s test was used to calculate the significance level for differences in expression between two conditions and the *p* values were corrected to control the false discovery rate (FDR) by the Benjamini–Hochberg procedure [50]. Due to their low fragment counts in the library, 2948 SNPs failed to pass the filter for the mean of normalized counts. At FDR less than 0.01, we classified the fragments to be PREs if their log_2_ (fold change) was greater than 0 or NREs if their log_2_ (fold change) was less than 0.

#### Identification of regulatory SNPs in PREs and NREs

For each of the 7725 SNPs passing the mean of normalized counts filter in DESeq2, the counts of reference and alternative alleles in the input library and the output library were calculated by SAMtools and bcftools [51, 52]. The counts were normalized by the SNP coverage for each library. The normalized reference and alternative allele counts from two replicates were pooled to increase statistical power. SNPs with pooled normalized reference or alternative allele counts less than 10 were excluded from further analysis. The effect size for each SNP was calculated as the fold change of allele ratios in the output library over the input library. Two-tailed Fisher’s exact test was applied to test the significance of differences in allele ratios between the two libraries. The *p* values were corrected using a Benjamini–Hochberg procedure to control the FDR to less than 10%.

#### Annotations used for epigenetic marker enrichments

For epigenetic marker enrichments with the screen hits, we obtained ChIP-seq data, DHS data, and TF binding data for HEK293 and HEK 293T cells from the ENCODE database. The ChIP-seq data from fetal kidney data were downloaded from the Roadmap Epigenomics Project (Additional file 3: Table S4 for data links) [53]. SNP-containing PREs and NREs were considered to overlap with the peaks if the SNP position was covered by the peak. Odds ratios were calculated as enrichment scores and Fisher’s exact test was applied to test the significance of the enrichment (Additional file 3: Table S1 for PRE and NRE enrichment, Table S2 for regulatory SNPs enrichment).

#### TF binding score analysis

The 500-bp SNP-containing regions were scanned using *Fimo* with human motif database HOCOMOCO v10 to predict TF binding [54, 55]. The predicted reference allele and alternative allele binding scores were calculated. Only those SNPs with either allele located in a predicted motif region and validated by the corresponding transcription factor binding from the ENCODE ChIP-seq peaks were considered. The delta score represents the binding score difference between the alternative allele and reference allele.

#### Topologically associating domain (TAD) viewer

The HiC data of interesting genomic regions were displayed using online tools (http://www.3dgenome.org) developed by the YUE lab.

#### eQTL analysis

We used a two-step linear regression model to perform the eQTL analysis in diffuse large B-cell lymphoma (DLBL) following the procedure of Li et al. [11]. Briefly, three factors were considered for gene expression level (*E*_*i*_): the somatic copy number variation (*C*_*i*_), the methylation of gene promoter region (*M*_*i*_), and the individual genotypes (*G*_*i*_). We downloaded these data for all the 48 DLBL cases from the TCGA project. A first step linear regression model was performed to normalize the methylation and the somatic copy number effect on gene expression, and the gene expression residual (*ε*_*i*_) was calculated:

$$ {E}_i={C}_i+{M}_i+{\varepsilon}_i $$

The genotype effect on gene expression level was determined by the second linear regression:

$$ {\varepsilon}_i={G}_i+{\omega}_i\left({\omega}_i\kern0.17em \mathrm{represents}\kern0.34em \mathrm{the}\kern0.34em \mathrm{random}\kern0.34em \mathrm{error}\right) $$

The analysis pipeline was applied to identify eQTL peaks in the following cancer types using TCGA datasets: breast invasive carcinoma (BRCA), colon adenocarcinoma (COAD), lung squamous cell carcinoma (LUSC), liver hepatocellular carcinoma (LIHC), prostate adenocarcinoma (PRAD), and stomach adenocarcinoma (STAD).

### Validation experiments and additional analysis

#### Luciferase reporter assays

Selected SNP-containing fragments were PCR amplified from HEK293T genomic DNA and cloned into the pGL4.23-SCP1 plasmid between the digestion sites for KpnI (NEB, R0142) and BglII (NEB, R0144). Multiple bacteria colonies were selected and grown individually for plasmid extraction. The genotype of each SNP in plasmids grown in each single colony was determined by Sanger sequencing. If only one genotype was detected, the construct containing the alternative allele was generated using a site-specific mutagenesis kit following the instructions of the manufacturer (NEB, E0554).

For the luciferase reporter assay, 2 × 10^5^ 293T Cells were plated in each well of a 24-well plate; 18 h later, cells were transfected with 20 ng of renilla vector along with 500 ng of pGL4.23-SCP1 firefly vector or pGL4.23-SCP1 vectors containing the selected fragments using Neofect DNA transfection reagent according to the manufacturer’s protocol. Twenty-four hours after transfection, cells were washed once with cold 1× PBS and the luciferase activities were measured with a Centro XS^3^ LB 960 Microplate Luminometer using Promega Dual Luciferase Assay kit (Promega, E1960). The firefly luciferase activity was normalized to renilla luciferase activity for each well. All the luciferase activity measurements were performed in triplicate for each condition. The Student’s *t*-test was applied to estimate the statistical significance of the difference in luciferase activities between the two conditions.

#### CRISPR interference experiments

For the selected enhancer region, sgRNAs were designed using online tools (http://crispr.mit.edu/) supplied by Feng Zhang’s Lab. The sgRNAs and the reverse complementary sequences were synthesized and annealed, then cloned into the lentiGuide-Puro plasmid (Addgene, #52963) and linearized by BsmBI (Thermo, ER0451) following the protocol as described by Zhang et al. [56, 57]. The sgRNA sequences are listed in Additional file 3: Table S5.

HEK 293T cells were transduced with lentivirus to stably express dCas9-KRAB [58]. Then the cells were seeded in a six-well plate and transfected with sgRNA plasmid using Lipofectamine® 2000 (Thermo, 11668019) at a density of 80%. After 72 h, cells were lysed by TRIzol Reagent (Thermo, 15596018).

#### qPCR for *ATF7IP* and *PDE4B* genes

cDNA synthesis from 1.5 μg total RNA was carried out in a 20 μL reaction using SuperScript® III First-Strand Synthesis SuperMix (Life Technologies, 18080400) with an oligo dT primer. cDNA (1 μL) was used as a template for qPCR analyses with FastStart SYBR Green MasterMix (Roche, 04673484001) with primers listed below. Relative gene expression was calculated using the ∆∆Ct method and the expression level was normalized by *GAPDH*. qPCR primers used were: *ATF7IP*-sense, GAGGAAGAAGAGCAAGTAATAC; *ATF7IP*-antisense, CATTGTCCATGTCTTCTGATT; *GAPDH*-sense, AGCACATCGCTCAGACAC; *GAPDH*-antisense, GCCCAATACGACCAAATCC. *PDE4B*-sense, ATGGTGTTAGCAACTGATATG; *PDE4B*-antisense, AGAACGCCTGAACTTGTA.

#### Differential gene expression analysis

For differential gene expression analysis, we performed Mann–Whitney U tests to evaluate the significance for the comparison of *PDE4B* expression levels between childhood acute lymphoblastic leukemia cases and non-leukemia controls. The microarray data were downloaded from Torsten Haferlach’s study [45]. R (version 3.2.2) was used to perform these statistical analyses and box plots were used to graphically display the distribution of gene expression between different groups.

## Additional files

Additional file 1: Supplementary figures. (DOCX 574 kb)

Additional file 2: Supplementary datasets. (XLSX 2496 kb)

Additional file 3: Supplementary tables. (DOCX 27 kb)

Additional file 4: Supplementary text for estimating the sensitivity of regulatory SNPs identification. (DOCX 23 kb)

## Abbreviations

DHS: DNase I hypersensitive site
eQTL: Expression quantitative trait loci
GWAS: Genome-wide association study
LD: Linkage disequilibrium
MPRA: Massively parallel reporter assay
NRE: Negative regulatory element
PRE: Positive regulatory element
SNP: Single nucleotide polymorphism
STARR-seq: Self-transcribing active regulatory region sequencing
TAD: Topologically associating domain

## References

[1] Welter D, MacArthur J, Morales J, Burdett T, Hall P, Junkins H, Klemm A, Flicek P, Manolio T, Hindorff L, Parkinson H (2014). The NHGRI GWAS Catalog, a curated resource of SNP-trait associations. Nucleic Acids Res.

[2] Ward LD, Kellis M (2012). Interpreting noncoding genetic variation in complex traits and human disease. Nat Biotechnol.

[3] Gao P, Wei GH. Genomic insight into the role of lncRNA in cancer susceptibility. Int J Mol Sci. 2017;18(6):1239. 10.3390/ijms18061239 PMC5486062 28598379

[4] Saunders MA, Liang H, Li WH (2007). Human polymorphism at microRNAs and microRNA target sites. Proc Natl Acad Sci U S A.

[5] Joehanes R, Zhang X, Huan T, Yao C, Ying SX, Nguyen QT, Demirkale CY, Feolo ML, Sharopova NR, Sturcke A (2017). Integrated genome-wide analysis of expression quantitative trait loci aids interpretation of genomic association studies. Genome Biol.

[6] Maurano MT, Humbert R, Rynes E, Thurman RE, Haugen E, Wang H, Reynolds AP, Sandstrom R, Qu H, Brody J (2012). Systematic localization of common disease-associated variation in regulatory DNA. Science.

[7] Schaub MA, Boyle AP, Kundaje A, Batzoglou S, Snyder M (2012). Linking disease associations with regulatory information in the human genome. Genome Res.

[8] Bernstein BE, Birney E, Dunham I, Green ED, Gunter C, Snyder M, ENCODE Project Consortium (2012). An integrated encyclopedia of DNA elements in the human genome. Nature.

[9] Nicolae DL, Gamazon E, Zhang W, Duan S, Dolan ME, Cox NJ (2010). Trait-associated SNPs are more likely to be eQTLs: annotation to enhance discovery from GWAS. PLoS Genet.

[10] Li Q, Stram A, Chen C, Kar S, Gayther S, Pharoah P, Haiman C, Stranger B, Kraft P, Freedman ML (2014). Expression QTL-based analyses reveal candidate causal genes and loci across five tumor types. Hum Mol Genet.

[11] Li Q, Seo JH, Stranger B, McKenna A, Pe’er I, Laframboise T, Brown M, Tyekucheva S, Freedman ML (2013). Integrative eQTL-based analyses reveal the biology of breast cancer risk loci. Cell.

[12] Huang Q, Whitington T, Gao P, Lindberg JF, Yang Y, Sun J, Vaisanen MR, Szulkin R, Annala M, Yan J (2014). A prostate cancer susceptibility allele at 6q22 increases RFX6 expression by modulating HOXB13 chromatin binding. Nat Genet.

[13] Oldridge DA, Wood AC, Weichert-Leahey N, Crimmins I, Sussman R, Winter C, McDaniel LD, Diamond M, Hart LS, Zhu S (2015). Genetic predisposition to neuroblastoma mediated by a LMO1 super-enhancer polymorphism. Nature.

[14] Yao L, Tak YG, Berman BP, Farnham PJ (2014). Functional annotation of colon cancer risk SNPs. Nat Commun.

[15] Whitington T, Gao P, Song W, Ross-Adams H, Lamb AD, Yang Y, Svezia I, Klevebring D, Mills IG, Karlsson R (2016). Gene regulatory mechanisms underpinning prostate cancer susceptibility. Nat Genet.

[16] Melnikov A, Murugan A, Zhang X, Tesileanu T, Wang L, Rogov P, Feizi S, Gnirke A, Callan CG, Kinney JB (2012). Systematic dissection and optimization of inducible enhancers in human cells using a massively parallel reporter assay. Nat Biotechnol.

[17] Patwardhan RP, Hiatt JB, Witten DM, Kim MJ, Smith RP, May D, Lee C, Andrie JM, Lee SI, Cooper GM (2012). Massively parallel functional dissection of mammalian enhancers in vivo. Nat Biotechnol.

[18] Arnold CD, Gerlach D, Stelzer C, Boryn LM, Rath M, Stark A (2013). Genome-wide quantitative enhancer activity maps identified by STARR-seq. Science.

[19] Vanhille L, Griffon A, Maqbool MA, Zacarias-Cabeza J, Dao LT, Fernandez N, Ballester B, Andrau JC, Spicuglia S (2015). High-throughput and quantitative assessment of enhancer activity in mammals by CapStarr-seq. Nat Commun.

[20] Ulirsch JC, Nandakumar SK, Wang L, Giani FC, Zhang X, Rogov P, Melnikov A, McDonel P, Do R, Mikkelsen TS, Sankaran VG (2016). Systematic functional dissection of common genetic variation affecting red blood cell traits. Cell.

[21] Tewhey R, Kotliar D, Park DS, Liu B, Winnicki S, Reilly SK, Andersen KG, Mikkelsen TS, Lander ES, Schaffner SF, Sabeti PC (2016). Direct identification of hundreds of expression-modulating variants using a multiplexed reporter assay. Cell.

[22] Zhou J. Functional genomic analysis of nuclear receptors in MCF7 cells. Proquest Dissertations Publishing; 2014. 3627912.

[23] Juven-Gershon T, Cheng S, Kadonaga JT (2006). Rational design of a super core promoter that enhances gene expression. Nat Methods.

[24] Liu Y. Nuclear receptor-mediated transcriptional regulation in prostate cancer cells. Proquest Dissertations Publishing; 2014. 3628087.

[25] Love MI, Huber W, Anders S (2014). Moderated estimation of fold change and dispersion for RNA-seq data with DESeq2. Genome Biol.

[26] Allis CD, Jenuwein T (2016). The molecular hallmarks of epigenetic control. Nat Rev Genet.

[27] Becker JS, Nicetto D, Zaret KS (2016). H3K9me3-dependent heterochromatin: barrier to cell fate changes. Trends Genet.

[28] Li B, Carey M, Workman JL (2007). The role of chromatin during transcription. Cell.

[29] Ong CT, Corces VG (2014). CTCF: an architectural protein bridging genome topology and function. Nat Rev Genet.

[30] Li W, Notani D, Rosenfeld MG (2016). Enhancers as non-coding RNA transcription units: recent insights and future perspectives. Nat Rev Genet.

[31] Corradin O, Saiakhova A, Akhtar-Zaidi B, Myeroff L, Willis J, Cowper-Sal lari R, Lupien M, Markowitz S, Scacheri PC (2014). Combinatorial effects of multiple enhancer variants in linkage disequilibrium dictate levels of gene expression to confer susceptibility to common traits. Genome Res.

[32] Low SK, Takahashi A, Ashikawa K, Inazawa J, Miki Y, Kubo M, Nakamura Y, Katagiri T (2013). Genome-wide association study of breast cancer in the Japanese population. PLoS One.

[33] Rao SS, Huntley MH, Durand NC, Stamenova EK, Bochkov ID, Robinson JT, Sanborn AL, Machol I, Omer AD, Lander ES, Aiden EL (2014). A 3D map of the human genome at kilobase resolution reveals principles of chromatin looping. Cell.

[34] Dixon JR, Selvaraj S, Yue F, Kim A, Li Y, Shen Y, Hu M, Liu JS, Ren B (2012). Topological domains in mammalian genomes identified by analysis of chromatin interactions. Nature.

[35] Pope BD, Ryba T, Dileep V, Yue F, Wu W, Denas O, Vera DL, Wang Y, Hansen RS, Canfield TK (2014). Topologically associating domains are stable units of replication-timing regulation. Nature.

[36] Gilbert LA, Larson MH, Morsut L, Liu Z, Brar GA, Torres SE, Stern-Ginossar N, Brandman O, Whitehead EH, Doudna JA (2013). CRISPR-mediated modular RNA-guided regulation of transcription in eukaryotes. Cell.

[37] Timms RT, Tchasovnikarova IA, Antrobus R, Dougan G, Lehner PJ (2016). ATF7IP-mediated stabilization of the histone methyltransferase SETDB1 is essential for heterochromatin formation by the HUSH complex. Cell Rep.

[38] Sun QY, Ding LW, Xiao JF, Chien W, Lim SL, Hattori N, Goodglick L, Chia D, Mah V, Alavi M (2015). SETDB1 accelerates tumourigenesis by regulating the WNT signalling pathway. J Pathol.

[39] Fei Q, Shang K, Zhang J, Chuai S, Kong D, Zhou T, Fu S, Liang Y, Li C, Chen Z (2015). Histone methyltransferase SETDB1 regulates liver cancer cell growth through methylation of p53. Nat Commun.

[40] Ceol CJ, Houvras Y, Jane-Valbuena J, Bilodeau S, Orlando DA, Battisti V, Fritsch L, Lin WM, Hollmann TJ, Ferre F (2011). The histone methyltransferase SETDB1 is recurrently amplified in melanoma and accelerates its onset. Nature.

[41] Yang JJ, Cheng C, Devidas M, Cao X, Campana D, Yang W, Fan Y, Neale G, Cox N, Scheet P (2012). Genome-wide association study identifies germline polymorphisms associated with relapse of childhood acute lymphoblastic leukemia. Blood.

[42] Nagy ZS, Ross JA, Rodriguez G, Balint BL, Szeles L, Nagy L, Kirken RA (2013). Genome wide mapping reveals PDE4B as an IL-2 induced STAT5 target gene in activated human PBMCs and lymphoid cancer cells. PLoS One.

[43] Suhasini AN, Wang L, Holder KN, Lin AP, Bhatnagar H, Kim SW, Moritz AW, Aguiar RC (2016). A phosphodiesterase 4B-dependent interplay between tumor cells and the microenvironment regulates angiogenesis in B-cell lymphoma. Leukemia.

[44] Smith PG, Wang F, Wilkinson KN, Savage KJ, Klein U, Neuberg DS, Bollag G, Shipp MA, Aguiar RC (2005). The phosphodiesterase PDE4B limits cAMP-associated PI3K/AKT-dependent apoptosis in diffuse large B-cell lymphoma. Blood.

[45] Haferlach T, Kohlmann A, Wieczorek L, Basso G, Kronnie GT, Bene MC, De Vos J, Hernandez JM, Hofmann WK, Mills KI (2010). Clinical utility of microarray-based gene expression profiling in the diagnosis and subclassification of leukemia: report from the International Microarray Innovations in Leukemia Study Group. J Clin Oncol.

[46] Farh KK, Marson A, Zhu J, Kleinewietfeld M, Housley WJ, Beik S, Shoresh N, Whitton H, Ryan RJ, Shishkin AA (2015). Genetic and epigenetic fine mapping of causal autoimmune disease variants. Nature.

[47] Vockley CM, Guo C, Majoros WH, Nodzenski M, Scholtens DM, Hayes MG, Lowe WL, Reddy TE (2015). Massively parallel quantification of the regulatory effects of noncoding genetic variation in a human cohort. Genome Res.

[48] Barrera LA, Vedenko A, Kurland JV, Rogers JM, Gisselbrecht SS, Rossin EJ, Woodard J, Mariani L, Kock KH, Inukai S (2016). Survey of variation in human transcription factors reveals prevalent DNA binding changes. Science.

[49] Li H, Durbin R (2009). Fast and accurate short read alignment with Burrows-Wheeler transform. Bioinformatics.

[50] Yoav Benjamini YH (1995). Controlling the false discovery rate: a practical and powerful approach to multiple testing. J R Stat Soc B Methodol.

[51] Li H (2011). A statistical framework for SNP calling, mutation discovery, association mapping and population genetical parameter estimation from sequencing data. Bioinformatics.

[52] Li H, Handsaker B, Wysoker A, Fennell T, Ruan J, Homer N, Marth G, Abecasis G, Durbin R, Genome Project Data Processing S (2009). The Sequence Alignment/Map format and SAMtools. Bioinformatics.

[53] Roadmap Epigenomics C, Kundaje A, Meuleman W, Ernst J, Bilenky M, Yen A, Heravi-Moussavi A, Kheradpour P, Zhang Z, Wang J (2015). Integrative analysis of 111 reference human epigenomes. Nature.

[54] Grant CE, Bailey TL, Noble WS (2011). FIMO: scanning for occurrences of a given motif. Bioinformatics.

[55] Kulakovskiy IV, Vorontsov IE, Yevshin IS, Soboleva AV, Kasianov AS, Ashoor H, Ba-Alawi W, Bajic VB, Medvedeva YA, Kolpakov FA, Makeev VJ (2016). HOCOMOCO: expansion and enhancement of the collection of transcription factor binding sites models. Nucleic Acids Res.

[56] Sanjana NE, Shalem O, Zhang F (2014). Improved vectors and genome-wide libraries for CRISPR screening. Nat Methods.

[57] Shalem O, Sanjana NE, Hartenian E, Shi X, Scott DA, Mikkelsen TS, Heckl D, Ebert BL, Root DE, Doench JG, Zhang F (2014). Genome-scale CRISPR-Cas9 knockout screening in human cells. Science.

[58] Larson MH, Gilbert LA, Wang X, Lim WA, Weissman JS, Qi LS (2013). CRISPR interference (CRISPRi) for sequence-specific control of gene expression. Nat Protoc.

