# Supplementary material for: Systematic identification of regulatory variants associated with cancer risk
Source: Genome Biol. 2017 Oct 23;18:194. doi: 10.1186/s13059-017-1322-z (PMC5651703; doi:10.1186/s13059-017-1322-z)
Supplement: Supplementary file 1 — Supplementary figures. (DOCX 574 kb) [file 13059_2017_1322_MOESM1_ESM.docx]

**Supplementary Figures**


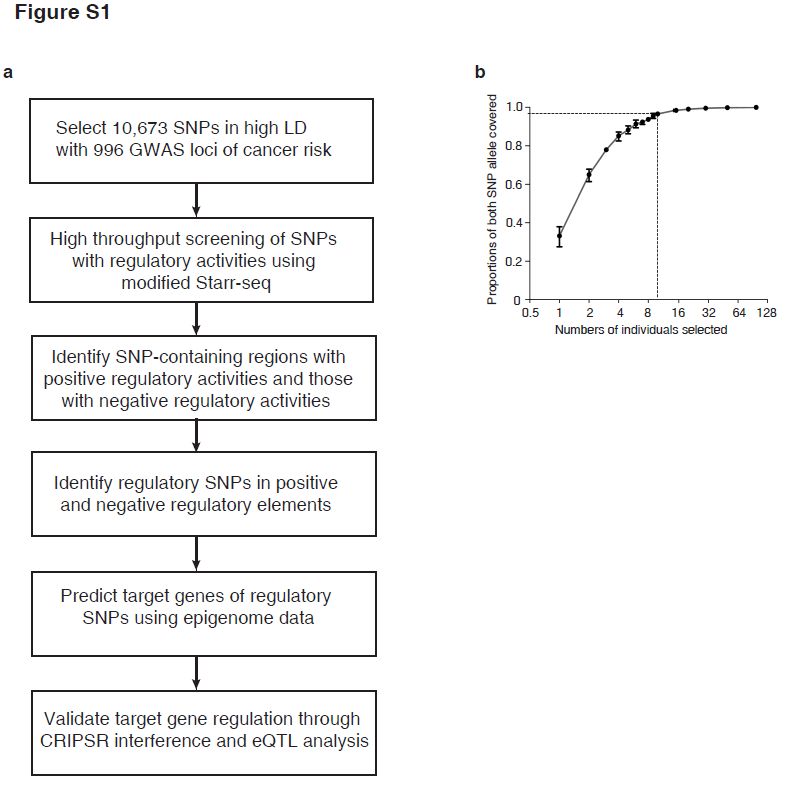


**Figure S1. a** Overall strategy for identifying regulatory variants associated with cancer risk. **b** Simulation of the proportions of both SNP alleles covered with different numbers of individuals selected using the data from 98 Chinese Han population individuals in the 1000 Genomes Project.


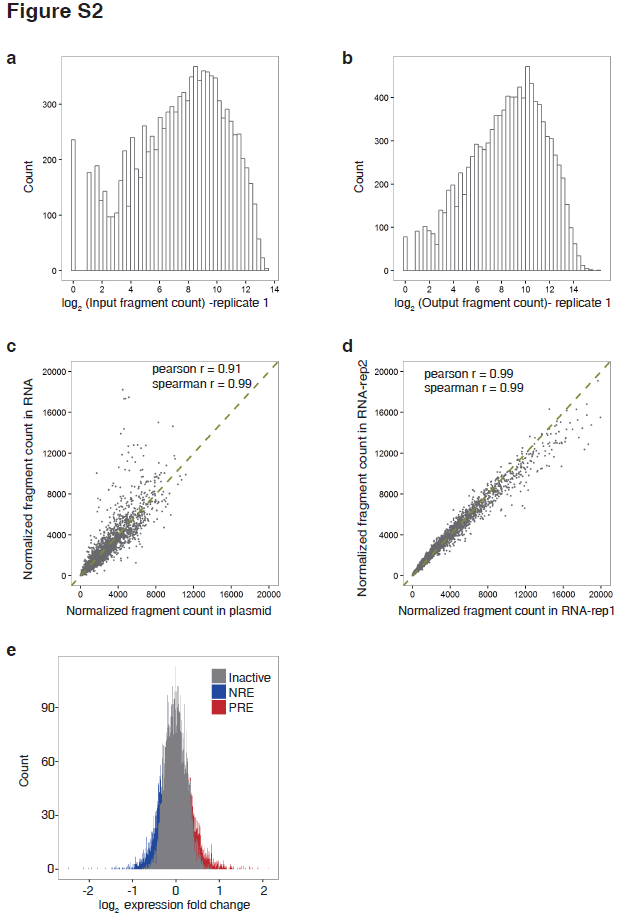


**Figure S2.** Technical performance of the screen. **a** Distribution of fragment counts for all the designed SNP-containing regions in the input library. **b** Distribution of fragments counts for all the designed SNP-containing regions in the output library. **c** Normalized fragment counts in the output library compared to the normalized fragment counts in the input library. **d** Correlation of normalized fragment counts between two transfection replicates of 293T cells. **e** Distribution of fold change in normalized read counts between the output RNA library and plasmid library for all the SNP containing regions tested. Identified PREs are in red and NREs are in blue.


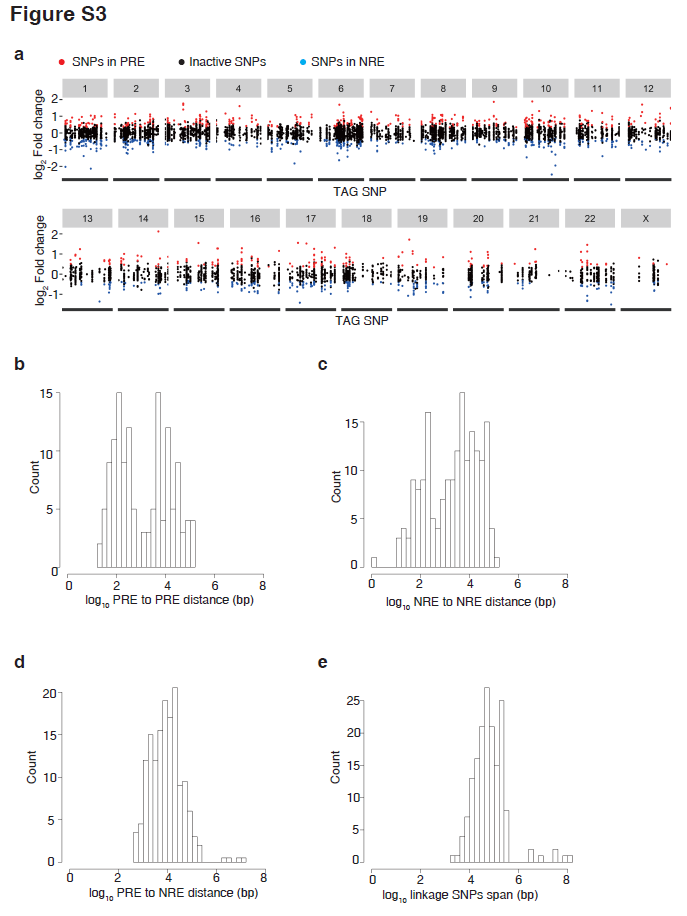


**Figure S3.** Distances between PREs and NREs in the same loci. **a** Distribution of SNPs across chromosomes. SNPs are plotted to the chromosome position of their tag SNPs reported in GWAS studies. SNPs in PREs are labeled red and SNPs in NREs are labeled blue. **b** Distribution of distances between PREs in the same loci. **b** Distribution of distances between NREs in the same loci. **c** Distribution of distances from each PRE to its closest NRE in the same loci. **d** Size distribution of each loci.


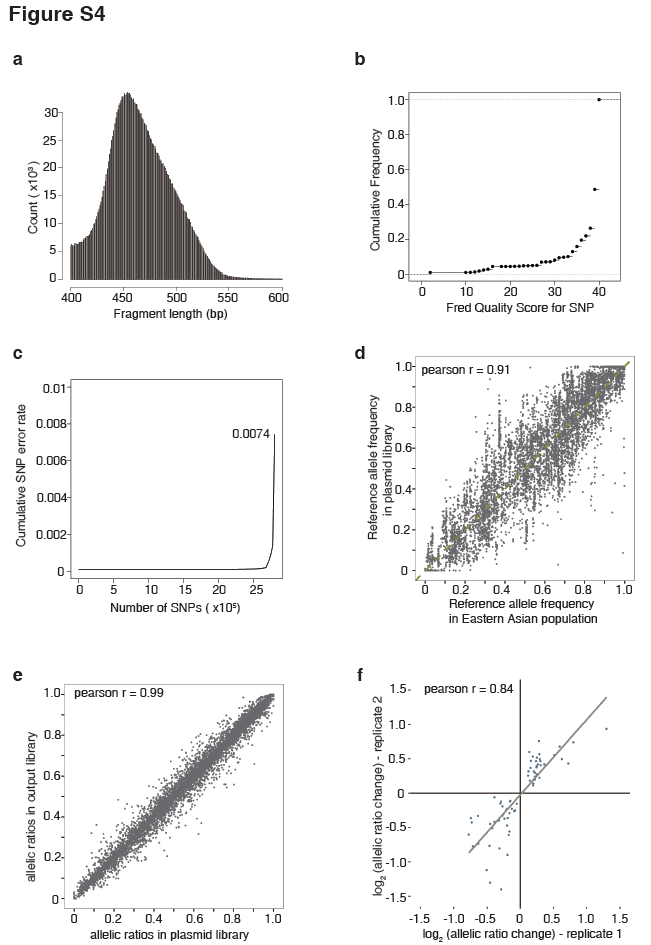


**Figure S4.** Tracking changes in allelic ratios in the screen. **a** Distribution of fragment size in the library. **b** Distribution of quality scores for all the SNP calls. **c** Cumulative sequencing error rates of calling SNPs. **d** Comparison of the allele frequency of each SNP in the input library to the allele frequency of each SNP in the Eastern Asian population. **e** Ratio of alternative allele against the reference allele in the input library compared to that in the output library. **f** Correlation of the calculated changes in allelic ratios between two replicates.


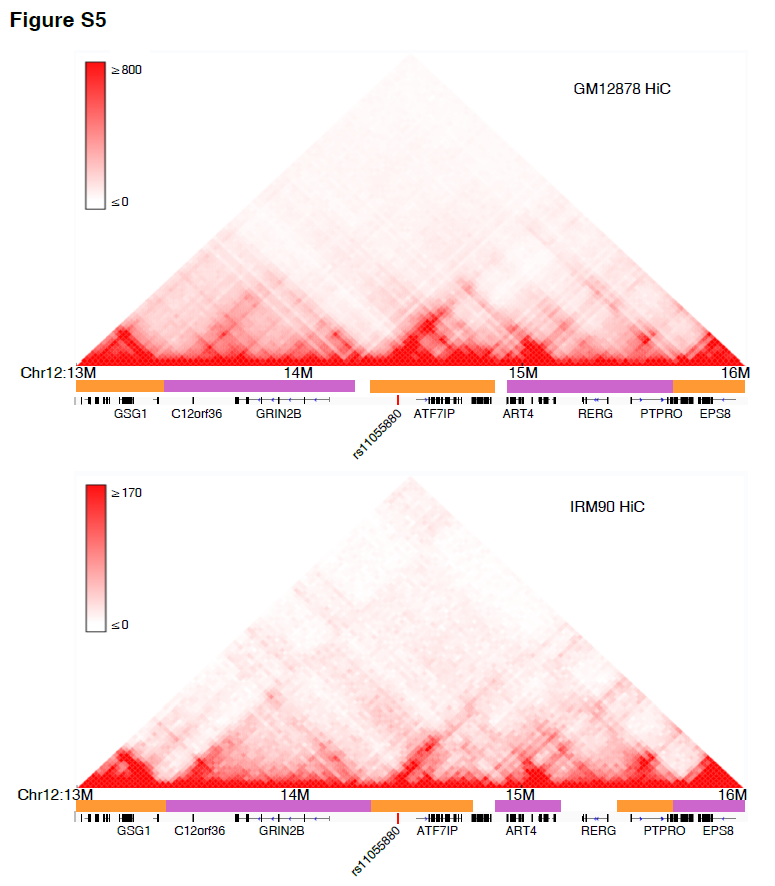


**Figure S5.** The chromatin interactions within chr12:13,000,000-16,000,000 identified by HiC mapping in GM12878 and IMR90 cell lines. Figure shows rs11055880 and *ATF7IP* gene are in one topological domain.
