# Supplementary material for: Systematic identification of regulatory variants associated with cancer risk
Source: Genome Biol. 2017 Oct 23;18:194. doi: 10.1186/s13059-017-1322-z (PMC5651703; doi:10.1186/s13059-017-1322-z)
Supplement: Supplementary file 3 — Supplementary tables. (DOCX 27 kb) [file 13059_2017_1322_MOESM3_ESM.docx]

**Supplementary Tables**

**Table S1.** The number of SNPs located in each epi-maker peaks.

| Epi-markers | PRE_SNPs | Inactive SNPs | log_2_OR | P value  (PRE) | NRE_SNPS | Inactive SNPs | log_2_OR | P value (NRE) |
| --- | --- | --- | --- | --- | --- | --- | --- | --- |
| POL2-HEK293 | 17 | 11 | 4.10 | 4.18 x10^-12^ | 0 | 11 | NA | 0.619 |
| CTCF-HEK293 | 6 | 48 | 0.47 | 0.451 | 14 | 48 | 1.30 | 0.006 |
| TF binding sites-ENCODE | 227 | 1538 | 0.71 | 9.09 x10^-9^ | 244 | 1538 | 0.42 | 3.03 x 10^-4^ |
| H3K27me3-Fetal kidney | 23 | 340 | -0.41 | 0.237 | 73 | 340 | 0.86 | 3.16 x10^-5^ |
| H3K4me1-Fetal kidney | 90 | 593 | 0.75 | 3.43 x10^-5^ | 73 | 593 | 0.05 | 0.743 |
| H3K36me3-Fetal kidney | 51 | 586 | -0.05 | 0.88 | 74 | 586 | 0.09 | 0.644 |
| H3K9me3-Fetal kidney | 1 | 54 | -2.28 | 0.086 | 7 | 54 | 0.13 | 0.834 |
| H3K9ac-Fetal kidney | 54 | 206 | 1.54 | 7.76 x10^-10^ | 57 | 206 | 1.22 | 3.21 x10^-7^ |
| H3K4me3-Fetal kidney | 52 | 198 | 1.55 | 1.47 x10^-9^ | 57 | 198 | 1.28 | 1.12 x10^-7^ |
| H3K4me3-HEK293 | 47 | 143 | 1.87 | 1.80 x10^-11^ | 53 | 143 | 1.64 | 3.13 x10^-10^ |
| DHS-HEK293T | 48 | 154 | 1.79 | 5.06 x10^-11^ | 55 | 154 | 1.59 | 3.50 x10^-10^ |
| DHS-Fetal kidney | 71 | 256 | 1.62 | 3.31 x10^-13^ | 70 | 256 | 1.21 | 3.47 x10^-8^ |
| Library SNPs | 758 | 6392 | NA | NA | 758 | 6392 | NA | NA |

**Table S2a.** The enrichment of regulatory SNPs within ENCODE TF binding clusters compared to inactive SNPs.

| Groups | TFBS | Total | OR | P value |
| --- | --- | --- | --- | --- |
| Regulatory NRE SNPs | 14 | 31 | 1.83 | 0.063 |
| Regulatory PRE SNPs | 21 | 39 | 2.18 | 5.48 x10^-3^ |
| other NRE SNPs | 206 | 643 | 1.30 | 2.79 x10^-3^ |
| other PRE SNPs | 176 | 463 | 1.54 | 7.57 x10^-6^ |
| Inactive SNPs | 1339 | 5424 | NA | NA |

**Table S2b.** The enrichment of regulatory SNPs within ENCODE TF binding clusters (Regulatory SNPs v.s. corresponding PRE/NRE SNPs).

| Groups | TFBS | Total | OR | P value |
| --- | --- | --- | --- | --- |
| Regulatory NRE SNPs | 14 | 31 | 1.409063 | 0.291 |
| other NRE SNPs | 206 | 643 |  |  |
| Regulatory PRE SNPs | 21 | 39 | 1.415773 | 0.231 |
| other PRE SNPs | 176 | 463 |  |  |

**Table S3.** The number of SNPs altering motif bindings.

| Groups | Inactive SNPs | PRE&NRE (not regulatory SNPs) | Regulatory SNPs |
| --- | --- | --- | --- |
| Delta score 0~1 | 19 | 8 | 0 |
| Delta score 1~3 | 21 | 7 | 1 |
| Delta score >3 | 69 | 35 | 6 |
| Library SNPs | 6392 | 1106 | 70 |

| ENCODE | http://hgdownload.cse.ucsc.edu/goldenPath/hg19/encodeDCC/wgEncodeAwgTfbsUniform/wgEncodeAwgTfbsSydhHek293Pol2UniPk.narrowPeak.gz |
| --- | --- |
|  | http://hgdownload.cse.ucsc.edu/goldenPath/hg19/encodeDCC/wgEncodeOpenChromDnase/wgEncodeOpenChromDnaseHek293tPk.narrowPeak.gz |
|  | http://hgdownload.cse.ucsc.edu/goldenPath/hg19/encodeDCC/wgEncodeUwHistone/wgEncodeUwHistoneHek293H3k4me3StdHotspotsRep1.broadPeak.gz |
|  | http://hgdownload.cse.ucsc.edu/goldenPath/hg19/encodeDCC/wgEncodeAwgTfbsUniform/wgEncodeAwgTfbsUwHek293CtcfUniPk.narrowPeak.gz |
|  | http://hgdownload.cse.ucsc.edu/goldenPath/hg19/database/wgEncodeRegTfbsClusteredV3.txt.gz |
|  |  |
| Roadmap project data | http://egg2.wustl.edu/roadmap/data/byFileType/peaks/consolidated/gappedPeak/E086-H3K4me1.gappedPeak.gz |
|  | http://egg2.wustl.edu/roadmap/data/byFileType/peaks/consolidated/gappedPeak/E086-H3K4me3.gappedPeak.gz |
|  | http://egg2.wustl.edu/roadmap/data/byFileType/peaks/consolidated/gappedPeak/E086-H3K9ac.gappedPeak.gz |
|  | http://egg2.wustl.edu/roadmap/data/byFileType/peaks/consolidated/gappedPeak/E086-H3K9me3.gappedPeak.gz |
|  | http://egg2.wustl.edu/roadmap/data/byFileType/peaks/consolidated/gappedPeak/E086-H3K27me3.gappedPeak.gz |
|  | http://egg2.wustl.edu/roadmap/data/byFileType/peaks/consolidated/gappedPeak/E086-H3K36me3.gappedPeak.gz |
|  | http://egg2.wustl.edu/roadmap/data/byFileType/peaks/consolidated/broadPeak/E086-DNase.hotspot.fdr0.01.broad.bed.gz |

**Table S4.** Data links for Figure 2 **c** and **d**.

| rs11055880 sgRNA2 sense | caccgTAATCTGCTTTATGGGGTCT |
| --- | --- |
| rs11055880 sgRNA2 antisense | aaacAGACCCCATAAAGCAGATTAc |
| rs11055880 sgRNA5 sense | caccgCCGCCCTGAACTGGATTTGG |
| rs11055880 sgRNA5 antisense | aaacCCAAATCCAGTTCAGGGCGGc |
| rs12142375 sgRNA2 sense | CACCGAGCAGGAAACTGCACTTGGA |
| rs12142375 sgRNA2 antisense | AAACTCCAAGTGCAGTTTCCTGCTC |
| rs12142375 sgRNA5 sense | CACCGGGTTTAGTTTAGTAATTTGC |
| rs12142375 sgRNA5 antisense | AAACGCAAATTACTAAACTAAACCC |
| NT sgRNA sense | caccggcgccaaacgtgccctgacg |
| NT sgRNA antisense | aaacCGTCAGGGCACGTTTGGCGCc |

**Table S5.** sgRNA sequences.
