# Supplementary material for: Systematic identification of regulatory variants associated with cancer risk
Source: Genome Biol. 2017 Oct 23;18:194. doi: 10.1186/s13059-017-1322-z (PMC5651703; doi:10.1186/s13059-017-1322-z)
Supplement: Supplementary file 4 — Supplementary text for estimating the sensitivity of regulatory SNPs identification. (DOCX 23 kb) [file 13059_2017_1322_MOESM4_ESM.docx]

**Supplementary Text**

**Estimating the sensitivity**

Due to the lack of a set of gold-standard positive and negative control regulatory variants, we estimated the sensitivity of our assay using two different strategies following previous work.

The first method assumed the causal variant of expression modulation falls into the eQTL peaks 34-41% of the time (Tewhey et al., 2016). We performed eQTL analysis using TCGA datasets of Breast Invasive Carcinoma (BRCA), Colon Adenocarcinoma (COAD), Lung Squamous Cell Carcinoma (LUSC), Liver Hepatocellular Carcinoma (LIHC), Prostate Adenocarcinoma (PRAD) and Stomach Adenocarcinoma (STAD). These datasets used SNP array for genotyping and we found 391 GWAS identified loci in the array was also included for our study. From the 391 tag SNP associated loci, we identified 56 eQTL peaks. The regulatory variants were found in 7.1% (4/56) of eQTL peaks, similar to the 8.6% value in Tewhey’s data for EUR population. Based on our luciferase assay, our predictive positive value (precision) for identifying the regulatory variants was 8/14=57%. Therefore we correctly identified about 4.0% of the eQTL peaks. Using the same assumption for population structure (34-41% in eQTL peaks), it corresponds to an estimate of 10%-12% in sensitivity, which is comparable to the estimated sensitivity of 9%-24% in Tewhey et al’s paper [1].

The second method assumed that there is only one causal variant for each GWAS association [2]. Under this assumption, we have included 451 GWAS associations for the test of regulatory variants and identified 70 regulatory variants from them. Then we used the formula “PPV*number of regulatory variants/total tested GWAS associations” to calculate the sensitivity. Our sensitivity was about 8.8%, slightly lower than an estimate of 14%-22% in their report.

1. Tewhey R, Kotliar D, Park DS, Liu B, Winnicki S, Reilly SK, Andersen KG, Mikkelsen TS, Lander ES, Schaffner SF, Sabeti PC: **Direct Identification of Hundreds of Expression-Modulating Variants using a Multiplexed Reporter Assay.** *Cell* 2016, **165:**1519-1529.

2. Ulirsch JC, Nandakumar SK, Wang L, Giani FC, Zhang X, Rogov P, Melnikov A, McDonel P, Do R, Mikkelsen TS, Sankaran VG: **Systematic Functional Dissection of Common Genetic Variation Affecting Red Blood Cell Traits.** *Cell* 2016, **165:**1530-1545.
